# Hsa-miR-25-3p Inhibition Sensitizes Patient-Derived Glioblastoma Cells to Temozolomide via β-catenin Downregulation

**DOI:** 10.1007/s10571-026-01750-6

**Published:** 2026-05-30

**Authors:** Katharina Richter, Hannah Markmann, Philipp Kaps, Annabell Wolff, Stefan Simm, Daniel Dubinski, Florian Gessler, Thomas M. Freiman, Timo Kirschstein, Christian Junghanss, Claudia Maletzki, Bjoern Schneider

**Affiliations:** 1 Department of Internal Medicine — Clinic and Polyclinic for Hematology, Hemostaseology, Oncology, Stem Cell Therapy and Palliative Medicine, Rostock University Medical Center, Schillingallee 70D, 18057 Rostock, Germany; 2 Institute of Pathology, Rostock University Medical Center, Rostock, Germany; 3 Institute of Bioinformatics, University Medicine Greifswald, Greifswald, Germany; 4 Institute for Bioanalysis, Coburg University of Applied Sciences and Arts, Coburg, Germany; 5 Department of Neurosurgery, Rostock University Medical Center, Rostock, Germany; 6 Oscar Langendorff Institute of Physiology, Rostock University Medical Center, Rostock, Germany; 7 Center of Transdisciplinary Neurosciences Rostock (CTNR), Rostock University Medical Center, Rostock, Germany

**Keywords:** Invasion, mRNA therapeutics, GBM stemness, Tumor suppressor gene

## Abstract

**Abstract:**

MicroRNAs (miRNAs) play pivotal roles in glioblastoma (GBM) progression and therapy resistance. Among them, miR-25-3p has emerged as a key oncogenic miRNA that promotes tumor growth, invasiveness, and resistance to temozolomide (TMZ). In this study, we profiled miRNA expression in primary GBM specimens (*n* = 50) stratified by *MGMT* methylation and *TP53* mutation status and assessed the functional impact of miR-25-3p inhibition in seven patient-derived GBM cell lines. Quantitative PCR analysis revealed upregulation of miR-135b in *MGMT*-methylated tumors and miR-10b in *TP53*-mutant cases. Both miR-25-3p and miR-10b were significantly elevated in 3D spheroid cultures compared to 2D monolayers. Notably, both miRNAs were secreted via tumor-derived extracellular vesicles, implicating a role in cell-cell communication. Inhibition of miR-25-3p in GBM cell lines consistently suppressed β-catenin and re-induced FBXW7 expression across all cases, correlating with inhibitor uptake. In four of seven cell lines, miR-25-3p inhibition enhanced TMZ sensitivity and reduced invasiveness, although the anti-invasive effect was not further potentiated by the addition of TMZ. In addition, RNA-Seq and methylome analyses revealed genetic and epigenetic reprograming toward a less aggressive, less invasive phenotype with reduced stemness potential. These findings highlight the interplay between tumor microenvironment and molecular heterogeneity in shaping miRNA dynamics in GBM. Collectively, our results identify miR-25-3p as a promising dual-action therapeutic target to mitigating both invasion and chemoresistance in GBM, warranting further translational investigation.

**Graphical Abstract:**

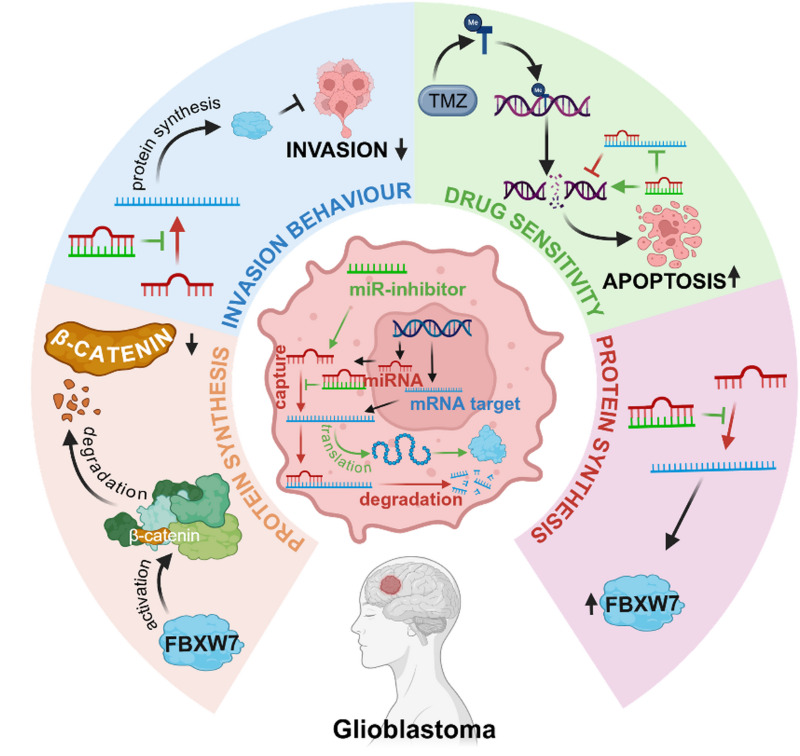

miRNA-mediated regulation of invasion, protein synthesis, and drug sensitivity in glioblastoma. Schematic overview of miRNA-mediated regulation of glioblastoma cell behavior. miRNAs modulate mRNA targets and protein synthesis, influencing β-catenin signaling, invasion capacity, and temozolomide sensitivity through FBXW7-associated pathways, ultimately affecting apoptosis.

## Introduction

In recent years, small RNA-based strategies to diagnose and treat human diseases have attracted increasing attention (Zhang et al. 2022; Kim and Croce 2023). More than 50 small interfering RNA therapeutics are currently undergoing evaluation in Phase I-III clinical trials (Zhang et al. 2021), with three already being approved by the Food and Drug Administration (Adams et al. 2018; Scott 2020; Scott and Keam 2021). Among these evolving RNA-based modalities, microRNAs (miRNAs) have attracted growing interest as both biomarkers and therapeutic targets. The importance of miRNAs was further underscored by the Nobel Prize in Physiology and Medicine 2024, awarded for the discovery and functional decoding of these regulatory miRNAs (Press release. NobelPrize.org. Nobel Prize Outreach 2025. n.d.). miRNAs are highly conserved, non-coding RNAs typically 22 nucleotides in length (Ambros 2004). Functionally analogous to siRNAs, they associate with Argonaute proteins to form a miRNA-induced silencing complex that binds to mRNAs at their target sites mostly located within the 3’-UTR through base complementarity, leading to translational repression (He et al. 2020). Consequently, miRNAs play a pivotal role in gene regulation at the translational level, influencing key cellular processes in both normal physiology and malignancy (Bartel 2004). Given their central role in different diseases, miRNAs have become a crucial point in cancer research and represent a promising frontier in RNA-based therapeutics.

Glioblastoma (GBM), classified as a WHO grade 4 tumor, is one of the most lethal cancers, with a 5-year survival rate of less than 10% (Ostrom et al. 2021). Therapeutic progress has been limited over the past three decades. The current standard of care includes maximal safe surgical resection (Davis 2016; Kotecha et al. 2023), followed by concomitant combined radio- and chemotherapy with temozolomide (TMZ) (Bei et al. 2010; Davis 2016; Tan et al. 2020; Kotecha et al. 2023). Additionally, tumor-treating fields—alternating electric fields that disrupt mitotic spindle formation—have been incorporated as an adjunctive, non-invasive treatment modality, demonstrating survival benefit in newly diagnosed GBM patients (Stupp et al. 2017; Jin et al. 2020; Kotecha et al. 2023). TMZ exerts its cytotoxic effects by methylating the O6 position of guanine, resulting in guanine- thymine mispairing during replication. This leads to TMZ induced DNA single- or double-strand breaks resulting in TMZ-mediated apoptosis (Stupp et al. 2001, 2005; Johannessen et al. 2008; Koukourakis et al. 2009; Singh et al. 2020). However, the clinical efficacy of TMZ is frequently limited by both intrinsic and acquired resistance mechanisms. A major factor contributing to poor treatment outcome is the pronounced inter- and intratumoral heterogeneity (Brennan et al. 2013; Patel et al. 2014; Darmanis et al. 2017; Neftel et al. 2019). Addressing this heterogeneity remains one of the main challenges for both preclinical and clinical approaches.

Although several miRNAs have been implicated in GBM biology, including miR-25-3p, important gaps remain regarding how miRNA dynamics are shaped by molecular heterogeneity, tumor microenvironmental context, and extracellular vesicle–mediated communication in primary patient-derived systems. While miR-25-3p has been described as an oncogenic miRNA in multiple cancers, such as colorectal cancer (Zeng et al. 2018), pancreatic cancer (Zhang et al. 2019), breast cancer (Chen et al. 2018) and liposarcoma (Sempere et al. 2021) and linked to proliferation, invasion, and TMZ resistance (Wang et al. 2021) in GBM through targeting of F-box/WD repeat-containing protein 7 (FBXW7) and Dickkopf-3 (DKK3) (Peng et al. 2019), its functional relevance across genetically stratified primary GBM specimens and patient-derived models has not been systematically evaluated. In particular, it remains unclear whether miR-25-3p inhibition consistently reprograms molecular signaling networks and reverses aggressive phenotypes in a context-dependent manner.

To address these gaps, we integrated miRNA profiling of primary GBM tissues stratified by MGMT methylation and TP53 mutation status with functional inhibition studies in seven patient-derived GBM cell lines cultured under both 2D and 3D conditions. Furthermore, we combined phenotypic assays with RNA sequencing and methylome analyses to assess transcriptional and epigenetic reprogramming following miR-25-3p inhibition.

Our results provide three key advances: (1) we demonstrate that miR-25-3p and miR-10b are enriched in 3D spheroid cultures and actively secreted via tumor-derived extracellular vesicles, highlighting microenvironment-dependent regulation and intercellular communication; (2) we show that miR-25-3p inhibition consistently suppresses β-catenin signaling and restores FBXW7 expression across genetically diverse patient-derived models, indicating a robust mechanistic axis; and (3) we reveal that miR-25-3p inhibition induces genetic and epigenetic reprogramming toward a less invasive, less stem-like phenotype, enhancing TMZ sensitivity in a subset of cases. Collectively, these findings position miR-25-3p not only as a biomarker candidate but as a functionally validated, dual-action therapeutic target capable of modulating both invasion and chemoresistance in molecularly heterogeneous GBM.

## Methods

### Patient Material

Formalin-fixed paraffin-embedded (FFPE) tumor tissues (*n* = 50) of patients with histologically proven primary GBM diagnosed from 07/2020 to 09/2023 (Table 1) were selected from the archival database of the Institute of Pathology, and clinical data were obtained from patients archives at the Rostock University Medical Center. Follow-up data were obtained from the regional Cancer Registries. The use of the patient material was approved by the local Ethics Committee (Rostock University Medical Center, Ethics Registration ID: A2018-0167), and conducted in accordance with the Declaration of Helsinki.

**Table 1:** Clinical and molecular characteristics of the GBM cohort (*n* = 50) included in miR analysis

Variable	*n* (%) or median (range)
Age at diagnosis (years)	68 (37–83)
Sex	
Male	28 (56)
Female	22 (44)
*MGMT* promoter methylation	
Methylated	25 (50)
Unmethylated	25 (50)
*TP53* mutation status	
Mutant	19 (38)
Wild-type	31 (62)
*IDH1* mutation status	
Mutant	2 (4)
Wild-type	48 (96)
Treatment received	
TMZ + RT	23 (46)
RT only	8 (16)
pRT	4 (8)
None	4 (8)
Other	4 (8)
n.a.	7 (14)
Overall survival (months)	11 (0–52)
Tumor localization	
Frontal lobe	17 (34)
Temporal lobe	13 (26)
Parietal lobe	7 (14)
Deep structures	7 (14)
n.a.	6 (12)

Most specimens were diagnosed as WHO grade 4 GBM with wild type IDH status. Two specimen were diagnosed following the old classification as WHO grade 4 GBM with IDH mutation

### 2D and 3D Cell Culture

Patient-derived GBM cell lines (GBM06, GBM15, and GBM26) were established from WHO grade 4 GBM specimens (Table 2) and cultured in 2D or 3D spheroids (Riess et al. 2022; Freitag et al. 2024). Intratumoral heterogeneity was addressed by establishing four morphological distinct clones (GBM24_2, _3, _4, _6) from the same tumor (Table 2), in some experiments, only one subclone was used and designated as GBM24 (referring to GBM24_4). Primary tumor samples were obtained from the Department of Neurosurgery, University Medical Center Rostock, Germany, with informed consent from all patients. The laboratory use of all the patient material was approved by the local Ethics Committee (Rostock University Medical Center, Ethics Registration ID: A2018-0167) and were conducted according the guidelines for the use of human materials, including the Declaration of Helsinki. Cell lines were cultured using Dulbecco’s Modified Eagle Medium/Nutrient Mixture F-12 (DMEM/F12) supplemented with 10% fetal calf serum, L-glutamine (6 mmol/l), and 1% penicillin/streptomycin (all from PAN-Biotech, Aidenbach, Germany). Cells were incubated at 37 °C in a humidified atmosphere containing 5% CO_2_.

**Table 2:** Clinical data of GBM cell lines

Lab-ID	Origin/location	MGMT status	Molecular profile
GBM06	m/71/temporal lobe	meth	*TP53* G244A (VAF: 99%); P72R (VAF: 100%); *PIK3CA* I391M (VAF: 30%)
GBM15	m/40/left parietal lobe	unmeth	*TP53* P72R (VAF: 99%)
GBM24	m/43/parietal lobe	unmeth	*PTEN* c.388 C > T p.R130* (VAF:100%), *RB1* stop (VAF: 100%)
GBM26	f/72/right temporal lobe	meth	*ATM* R2443* (VAF: 52%)

All specimens were diagnosed as WHO grade 4 GBM with a wild-type IDH status. *m* male, *f* female, *meth* methylated, *unmeth* unmethylated, *VAF* Variant Allele Frequency

### RNA Extraction

**Tumor tissue**: ten-micrometer sections of FFPE tissue blocks were used to extract RNA using the miRNeasy^®^ FFPE Kit (Qiagen, Hilden, Germany) following the manufacturer’s instructions.

**Cell culture**: 5 × 10^5^ cells or eight spheroids were used to extract RNA using the miRNeasy^®^ Mini Kit (Qiagen), following the manufacturer’s instructions.

**Extracellular vesicles (EV)**: between 1.5 and 3 × 10^10^ EVs in 200 µl PBS were used to extract RNA using the miRNeasy^®^ Plasma and Serum Kit (Qiagen).

### microRNA‑specific Reverse Transcription and Quantitative Real-Time-PCR

The initial miR-expression profiling was performed using the nCounter Human v3 miRNA assay (Nanostring, Seattle, WA, USA) comprising 827 human miRNAs and internal reference controls. The selection of the final miRs was regarding significance differential expression between clinical and pathological subgroups, high abundance in GBM and/or mentioning in the literature as important for glioblastoma development or treatment resistance. Expression analysis of miR-10b-5p, miR-25-3p, miR-135b-5p, miR-135a-5p, miR-151a-5p, miR-129-3p, miR-129-5p, and miR-34c-5p was performed by qPCR using miRNA-specific TaqMan Assays (Applied Biosystems, Darmstadt, Germany) with RNU6B as endogenous control (Applied Biosystems). Reverse transcription of the eight miRNAs and RNU6B was performed with the TaqMan MicroRNA Reverse Transcription Kit (Applied Biosystems) according to the manufacturer’s protocol modified as follows: the reaction volume was scaled up to 30 µl and 30 ng RNA were used as template. The reverse transcription was carried out as a multiplex reaction, containing the RT-primer for all miRNAs and the control in one reaction. The subsequent qPCR reactions were set up according to the manufacturer’s protocol using TaqMan Universal PCR Master Mix II, No UNG (Applied Biosystems) and the miRNA-specific primer/probe mixes. The runs were performed on a StepOne Plus Real-time PCR system (Applied Biosystems) and the data analyzed using the StepOne Software v2.1 (Applied Biosystems). Relative expression against RNU6B (fold change) was calculated using the ΔCt-algorithm.

### Isolation of EVs and Nanoparticle Tracking Analysis

Cells were cultured until 80% confluence, followed by a complete medium exchange with DMEM/F-12. After 48 h in an incubator, 6 ml of conditioned DMEM/F-12 medium was collected, aliquoted in 2 ml portions and stored at − 80 °C. For EV isolation, thawed supernatants were centrifuged at 16,000 *× g* for 10 min to remove cellular debris. Approximately 90% of the clarified supernatant was divided between two Ultra-Clear centrifuge tubes (Beckman Coulter, Brea, California, USA), supplemented with 9 ml of 1× PBS per tube, and subjected to ultracentrifugation at 120,000 *× g* for 2 h to pellet the EVs. The supernatant was discarded, and the EV pellets were suspended in 250 µl of 1× PBS. EVs were stored at − 80 °C until further use. This was performed for *n* = 4 cell lines with three independent experiments each with three technical replicates.

For quantification, EVs were diluted in PBS and analyzed using a NanoSight^®^ LM10 (Malvern Panalytical, Malvern, United Kingdom, Software: Nanoparticle Tracking Analysis 3.3). Five 30 s video recordings were acquired per sample, with a thermometer attached to monitor temperature. Mean size and concentration were calculated, and PBS background was subtracted.

### Application of miRNA-inhibitors

Cells were seeded at defined densities depending on the plate format (18-well µ-slides: 500 cells/well, 96-well plates: 500 cell/well, and 24-well plates: 5,000 cells/well). After 24 h of incubation, the supernatant was replaced with DMEM/F-12 medium containing fluorescence labeled miRCURY LNA miR Power Inhibitor (miR-25-3p or inhibitor control, 0.1 µM; Qiagen). This treatment was repeated after five days, and cells were processed for further experiments five days later. The binding of the miRNA-inhibitor was documented by fluorescence microscopy (Axiovert A.1, Zeiss, Jena, Germany; filter: Colibri 5, Zeiss; objective lens: LD A-Plan 5x/0,15 Ph1 M27, Zeiss; software: Zen 3.0, Zeiss) and evaluated using ImageJ (v1.54p, RRID: SCR_003070). This was performed for *n* = 7 cell lines with three independent experiments each with three technical replicates.

### TMZ Treatment Protocol

Following miRNA-inhibitor treatment, cells were exposed to TMZ (MSD, Haar, Germany, 10 µM, 2 × 72 h). Thereafter, biomass of 2D cell culture was determined using crystal violet staining: cells were washed with PBS, stained for 10 min with 0.2% crystal violet, washed twice with PBS, incubated with 1% SDS, and absorbance was read at 570/620 nm (Infinite 200 Pro, Tecan Group AG, Männedorf, Switzerland). Relative biomass was calculated versus miRNA-control. For 3D spheroids, viability was assessed with the CellTiter 3D Glo Kit (Promega, Walldorf, Germany), and luminescence was measured on the Infinite 200 Pro with 1 s integration. This was performed for *n* = 2–7 cell lines with three independent experiments each with three technical replicates.

### Immunfluorescence Staining

Following a 10-day miRNA-inhibitor cycle, cells seeded into 18-well µ-slides were washed with 100 µl/well PBS and incubated in 2% PFA for 15 min. After washing with PBS, cells were permeabilized and blocked in 0.5% Triton X-100/2% BSA for 1 h. Cells were then incubated overnight at 4 °C with primary antibodies diluted in blocking solution (1% BSA, 0.5% Triton X-100 in PBS): PE anti-β-catenin 1 (1:100; 0.05 µg/ml; RRID: AB_2832862, BioLegend, San Diego, California, USA), anti-FBXW7 (1:200; 5 µg/ml; RRID: AB_2724297, Thermo Fisher Scientific, Darmstadt, Germany, 1:1750) and anti-DKK3 (1:200; 5 µg/ml; RRID: AB_3093755, Thermo Fisher Scientific). After washing the cells three times with PBS, cells were incubated for 60 min at room temperature with secondary antibody Alexa Fluor^®^ 647 (1:250; 2 µg/ml; RRID: AB_2535804, Thermo Fisher Scientific) and Alexa Fluor^®^ 546 (1:500; 4 µg/ml; AB_2737024, Thermo Fisher Scientific). Nuclei were stained with DAPI (stock: 1.75 mg/ml; Thermo Fisher Scientific, 1:1750) for 2 min, and cells were analyzed using fluorescence microscopy (objective lens: LD A-Plan 5x/0,15 Ph1 M27) and quantified with ImageJ. This was performed for *n* = 4 cell lines with three independent experiments each with three technical replicates.

### Cell Stress Analyzes

Cells were seeded into 18-well µ-slides, treated with miRNA-inhibitor, and then stained with MitoTracker^®^ Red CMXRos (1:50,000; 20 nM; Cell Signaling Technology, Cambridge, UK) and ER-Tracker™ Blue-White DPX (1:1000; 1 µM; Cell Signaling Technology) in DMEM/F12 for 35 min at 37 °C in an incubator. Next, the cells were washed twice with PBS and analyzed immediately via fluorescence microscopy (objective lens: LD A-Plan 5x/0,15 Ph1 M27) in PBS. This was performed for *n* = 2 cell lines with three independent experiments each with three technical replicates.

### 3D Spheroid Invasion Assay

Cells treated with miRNA-inhibitors were used to generate spheroids, which after three to four days were embedded in a mixture of matrigel (Corning, Corning, New York, USA), the respective treatment, and 1% EGF. After 1 h incubation to allow gelation, medium containing the respective treatment was added, and the plate was stored in an incubator at 37 °C.The invasion behavior of the spheroids into the matrix was monitored on days 1, 4, and 7 using light microscopy (objective lens: LD A-Plan 40x/0,55 Ph1 M27). This was performed for *n* = 7 cell lines with three independent experiments each with three technical replicates.

### RNA Sequencing and Analysis

Cells were treated twice for five days with miRNA-inhibitors and 5 × 10^5^ cells were pooled per condition. Total RNA was isolated using RNeasy Mini Kit (Qiagen) with DNase treatment according to the manufacturer’s instruction, and samples with OD260/280 ≥ 2.0 and OD260/230 ≥ 2.0 were sequenced after polyA enrichment on the Illumina PE150 sequencing platform with 30 Mio reads/sample (paired end analysis), service provided by Novogene. Bioinformatical analyses including counting, mapping, and quality control were provided by Novogene. Differentially expressed genes (DEG) between miRNA-control and miRNA-inhibitor treated condition was performed via DeSeq2 using R scripts based on xlsx-files. Gene Ontology (GO) enrichment analyses were performed using the web tool INDRA GO. Volcano plot was created in R studio (version 4.4.2, RRID: SCR_000432), and STRING networks were visualized using cytoscape (version 3.10.4, RRID: SCR_003032). Gene set enrichment analysis (GSEA) was performed using fgsea R-package (v1.16.0; Korotkevich et al. 2016) and visualized in R studio. This was performed for one cell lines with three biological replicates.

### Human Whole Genome Bisulfite Sequencing and Analysis

Cells were treated twice for five days with miRNA-inhibitors and 5 × 10^5^ cells were pooled per condition. Total DNA was isolated using the Maxwell^®^ CSC Genomic DNA Kit (Promega) according to the manufacturer’s instruction, and samples with OD260/280 ≥ 1.8 and OD260/230 ≥ 2.0 were sequenced on the Illumina PE150 sequencing platform, service provided by Novogene. Bioinformatic analysis was performed using Galaxy Framework (Europe). First the reads were mapped on the hg38 genome using BWA-Mem2 (*BWA-Mem2*, no date) with default settings. Next the data were further processed using BWAmeth (*BWAmeth*, no date) with default parameters. As the next step MethylDackel (MethylDackel n.d.; Tanić et al. 2022) was used to count CpG, CHG and CHH positions. These data were analyzed using python scripts based on processing bedgraph-files. The information were included for DEGs between miRNA-control and miRNA-inhibitor treated condition and visualized with volcano plots created in R studio. This was performed for *n* = 2 cell lines with three biological replicates.

### Blinding

All experiments were carried out with experimenters blinded to the experimental protocol to minimize bias. The statistical analyses themselves were not performed in a blinded manner, still, the performed technical and biological replicates ensures that the results remain unbiased and reliable.

### Statistics

Statistical analyses and graphs were generated with GraphPad Prism 10.5.0 (GraphPad Software, San Diego, California, USA; RRID: SCR_002798). Each data set was tested for normality using the Shapiro-Wilk test. After proving the assumption of normality, one-way ANOVA (Dunnett’s or Holm-Sidak’s multiple comparisons test), two-way ANOVA (Tukey’s multiple comparisons test), or t-test (paired or unpaired) was performed. If the normality test failed, the Kruskal–Wallis (Dunnett’s multiple comparisons test) was performed. Statistical tests and sample sizes are reported in the figure legends. Multiple-Test correction was performed using FDR and data are presented as mean ± SD, and adjusted *p* < 0.05 was considered significant.

### Code Availability

The amplicon sequencing data of this study are available in the Gene Expression Omnibus repository at https://www.ncbi.nlm.nih.gov/sra (Accession No. PRJNA1439168 and PRJNA1439717).

## Results

### miRNA Expression Levels Reveal High Degree of Heterogeneity

**Fig. 1:**
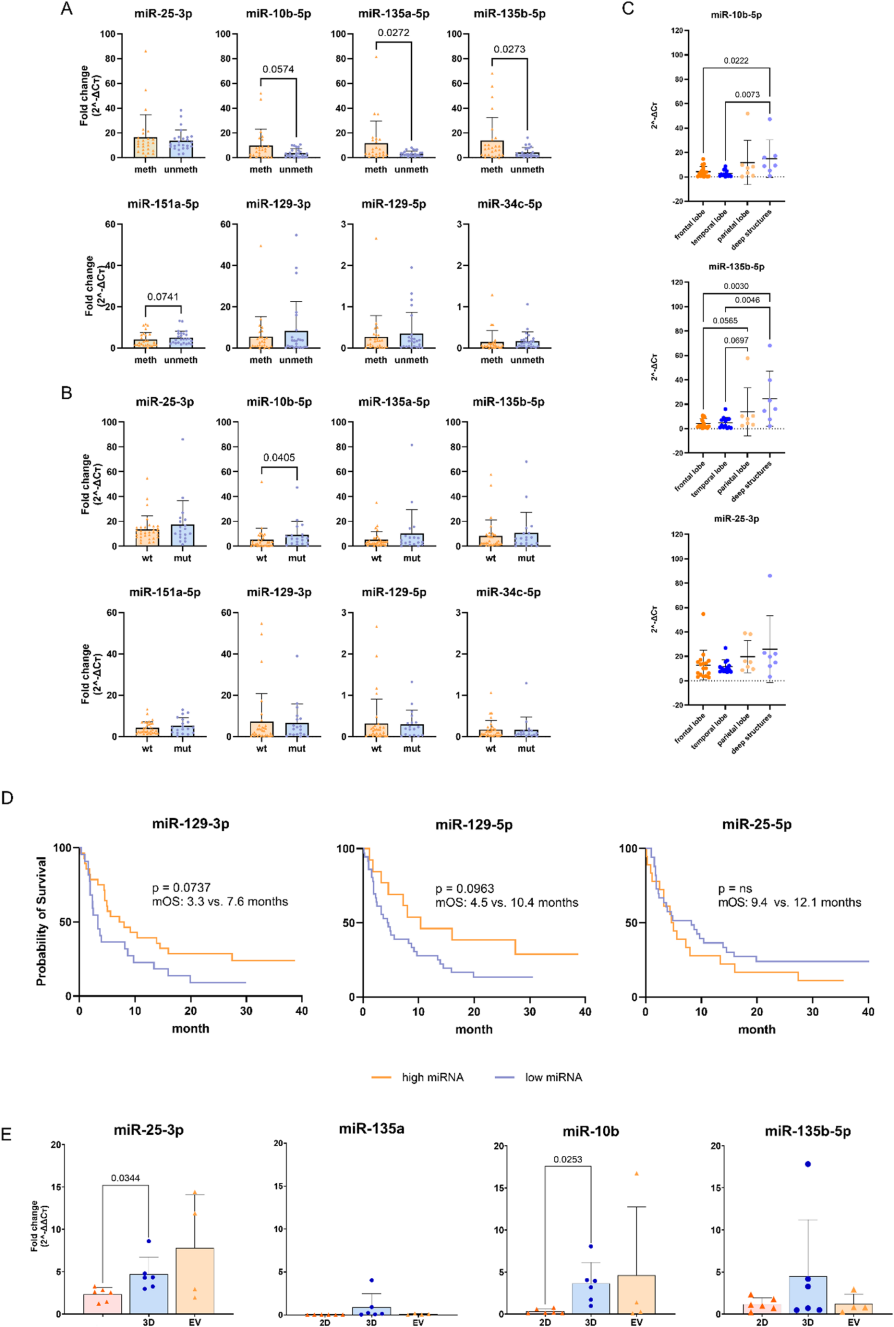
Differential expression of selected miRs in relation to MGMT methylation status, TP53 mutation status, patient survival, and culture conditions. **A** Graphs represent relative expression levels of eight selected miRs in FFPE tumor tissue stratified by MGMT promoter methylation status (meth = methylated, unmeth = unmethylated) analyzed by qPCR and quantified relative to RNU6B, which served as endogenous control, given as fold change (2^-∆CT). Mean + SD, *n* = 50 (meth = 25, unmeth = 25). Mann Whitney test. **B** Graphs represent miR expression levels in tumors grouped by TP53 mutation status (mut = mutated, wt = wild-type), analyzed by qPCR and quantified relative to RNU6B, which served as endogenous control, given as fold change (2^-∆CT). Mean + SD, *n* = 50 (wt = 31, mut = 19). Mann Whitney test. **C** Graphs represent relative expression levels of miR-10b-5p (top), miR-135b-5p (middle), and miR-25-3p (bottom) in FFPE tumor tissue stratified by localization of the tumor analyzed by qPCR and quantified relative to RNU6B, which served as endogenous control, given as fold change (2^-∆CT). Mean + SD, *n* = 44 (frontal lobe = 17, temporal lobe = 13, parietal lobe = 7, and deep structures = 7). Kruskal-Wallis test. **D** Kaplan-Meier survival analysis for high/low expression of miR-129-3p (left), miR-129-5p (middle), and miR-25-3p (right). *n* = 50, left: low = 22, high = 28; middle: low = 37, high = 13; right: low = 32, high = 18. log-rank test. **E** Graphs represent miRs (miR-25-3p, miR-10b-5p, miR-135a-5p, miR-135b-5p) in 3D spheroid cultures compared to 2D monolayer cultures and EVs derived from 2D cell culture, normalized to expression levels in primary tumors given as fold change (2^-ΔΔCT). Mean + SD, *n* = 4–6 cell lines with three independent experiments with three technical replicates. Paired t test

We first analyzed the expression of eight miRNAs in FFPE tumor tissues. These miRNAs were identified through a NanoString-based prescreening of 827 miRNAs and were chosen based on significantly differential expression across clinical and pathological subgroups, high abundance in GBM, and/or prior evidence in the literature supporting their relevance to progression or treatment resistance. Based on this, GBM cases were stratified by *MGMT* promotor methylation and *TP53* mutation status (Fig. 1A, B). In *MGMT*-methylated samples, miR-135a-5p and miR-135b-5p were significantly upregulated (*p* < 0.05), with miR-10b-5p and miR-25-3p also showing higher expression in methylated cases, though not statistically significant. In contrast, miR-151a-5p, miR-129-3p, and miR-129-5p displayed higher expression in *MGMT*-unmethylated samples. miR-34c-5p expression was unaffected by *MGMT* methylation status (Fig. 1A).

Regarding *TP53* mutation status, miR-10b-5p was significantly upregulated in *TP53*-mutated tumors (*p* < 0.05). Trends toward increased expression were also observed for miR-135a-5p, miR-135b-5p, miR-151a-5p, and miR-25-3p, while miR-129-3p and miR-129-5p showed no consistent pattern. Again, miR-34c-5p expression remained unaffected regardless of *TP53* status (Fig. 1B). In addition, we examined the miRNA expression in relation to tumor localization. Significant differences in the expression of both miR-10b-5p and miR-135b-5p were observed, with higher expression overall and a more diverse expression pattern of the two miRNAs seen in tumors located in the parietal lobe and deep structures, while tumors in the frontal and temporal lobes showed closer grouping and generally reduced expression compared to the other two regions (Fig. 1C). The expression levels of miR-25-3p, despite consistently high, did not significantly differ between brain areas (Fig. 1C), highlighting the overall importance of this oncogenic miRNA. To assess potential clinical relevance, we examined the association between miRNA expression and overall survival. Kaplan–Meier analysis showed a moderate positive correlation between higher expression of miR-129-3p (*r* = 0.41, *p* = 0.07) and miR-129-5p (*r* = 0.38, *p* = 0.09 and extended survival, as well as a low correlation between lower expression of miR-25-3p (*r* = 0.23, p = ns) and extended survival (Fig. 1D), suggesting potential prognostic value.

Next, we studied the expression of four selected miRNAs (miR-25-3p, miR-129-3p, miR-10b-5p, and miR-135a-5p) under physiological conditions using both 2D monolayer cultures and 3D spheroid models. Relative expression changes were calculated against primary tumor tissue using the 2^-ΔΔCT method. All four miRNAs showed increased expression in 3D cultures compared to 2D, with significant upregulation observed for miR-25-3p and miR-10b-5p (*p* < 0.05) (Fig. 1E).

EVs derived from 2D monolayer cultures revealed pronounced heterogeneity in miRNA expression across cell lines, particularly for miR-25-3p and miR-10b-5p, with some cell lines exceeding the miRNA-expression level of the other models. In contrast, both miR-135a and miR-135b displayed expression patterns closely mirroring those of their respective 2D cell culture. Hence, tumor-derived EVs consistently harbored the respective miRNAs, implicating a functional role in GBM. Given its consistent upregulation across methylation, *TP53* status, and culture conditions—as well as its biological relevance—we ultimately choose miR-25-3p for further functional studies.

### miR-25-3p Inhibition Increases TMZ Sensitivity

**Fig. 2:**
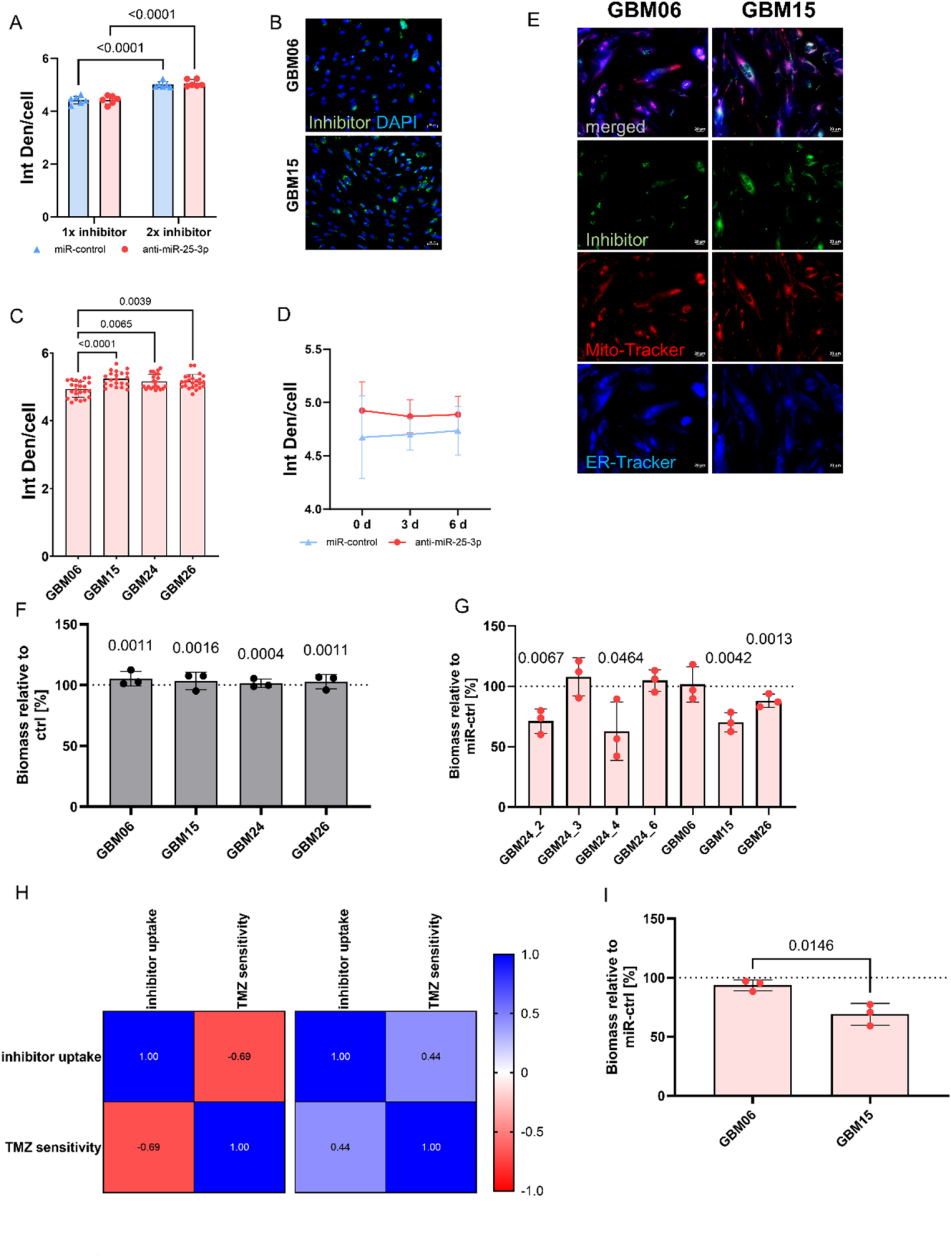
Inhibition of miR-25-3p enhances TMZ sensitivity in patient-derived glioblastoma cell lines. **A** Intracellular uptake of miR-25-3p inhibitor after single (1×) or repeated (2×) transfection over a 10-day period. Mean ± SD, *n* = 7 cell lines with three independent experiments with three technical replicates. Paired t test. **B** Representative fluorescence images of miR-25-3p inhibitor uptake in GBM06 and GBM15 after repeated application. Scale bar = 20 μm. **C** Quantitative miR-25-3p inhibitor uptake for GBM06, GBM15, GBM24_4, GBM26. Mean ± SD, *n* = 4 independent experiments with three technical replicates. One-way ANOVA. **D** Graph represents stability of miR-25-3p inhibitor binding over 6 days post-transfection (2 × 5 d) after removing all unbound inhibitors. Mean ± SD, *n* = 3 independent experiments with three technical replicates. **E** Representative fluorescence images of GBM15 and GBM06 stained with Mito- and ER-Tracker after miR-inhibitor treatment for 2 × 5 d. Scale bar = 20 μm. **F** Quantification of biomass of four 2D cell lines after TMZ treatment for 2 × 72 h, in relation to cells treated with DMSO. Mean ± SD, *n* = 3 independent experiments with three technical replicates. t-test. **G** Quantification of biomass of seven 2D cell lines after TMZ treatment for 2 × 72 h, following a miR-inhibitor application (2 × 5 days), in relation to cells incubated with miR-control. Mean ± SD, *n* = 3 independent experiments with three technical replicates. t-test. **H** Pearson Correlation between inhibitor uptake and TMZ sensitivity for TMZ resistant cell line (left) and TMZ sensitive cell lines (right). **I** Quantification of biomass of two 3D spheroid cell lines after TMZ treatment for 2 × 72 h, following a miR-inhibitor application (2 × 5 days), in relation to cells incubated with miR-control. Mean ± SD, *n* = 3 independent experiments with three technical replicates. t-test

Given that association between elevated miR-25-3p expression and TMZ resistance, we investigated the effects of miR-25-3p inhibition using a sequence-specific miRNA-inhibitor. To evaluate delivery efficiency, we compared single versus repeated applications of the miR-25-3p inhibitor over a 10-day period. Repeated transfection (twice) significantly increased intracellular uptake compared to a single application (*p* < 0.0001), as measured by fluorescence intensity (Fig. 2A, B) Significant differences were also observed between the cell lines (Fig. 2C). Furthermore, the inhibitor remained stably bound to its target throughout the observation period (Fig. 2D). To rule out off-target toxicity from the miRNA-inhibitor, we assessed cellular stress levels using Mito- and ER-Tracker staining. No signs of mitochondrial or endoplasmic reticulum stress were detected (Fig. 2E), confirming that the inhibitor was well tolerated at the applied concentrations.

We next investigated whether miR-25-3p inhibition could sensitize intrinsically TMZ-resistant GBM cells to this drug (Fig. 2F). Patient-derived GBM cell lines were treated with TMZ following miR-25-3p inhibition. In two of the first three cell lines (GBM15 and GBM26), miR-25-3p inhibition significantly enhanced TMZ sensitivity, resulting in up to 40% reduction in biomass compared to miRNA-control-treated cells (*p* < 0.05; Fig. 2G). One cell line (GBM06) did not respond to the treatment, which correlated with the lowest observed inhibitor uptake (Fig. 2C, H). Importantly, *MGMT* promoter methylation status did not predict response to miR-25-3p inhibition, as both responsive and non-responsive cell lines included methylated and unmethylated cases (Fig. 2G). Moreover, the uptake intensity correlated with sensitization to TMZ (Fig. 2H). These findings were consistent in 3D cell culture models: GBM15 displayed increased TMZ sensitivity following miR-25-3p inhibition, while GBM06 remained resistant (Fig. 2I). To further investigate intratumoral heterogeneity, we analyzed four cell lines derived from the same primary tumor (GBM24_2, GBM24_3, GBM24_4, and GBM24_6). Despite their shared origin, only two responded to TMZ more effectively after miR-25-3p inhibition, underscoring functional diversity in miRNA dependency among subclones within a single tumor (Fig. 2G). Cumulatively, these data indicate a direct link between miR-25-3p inhibition and TMZ sensitivity.

### Correlation Between miRNA-inhibitor Uptake, β-catenin Downregulation and FBXW7 Upregulation

**Fig. 3:**
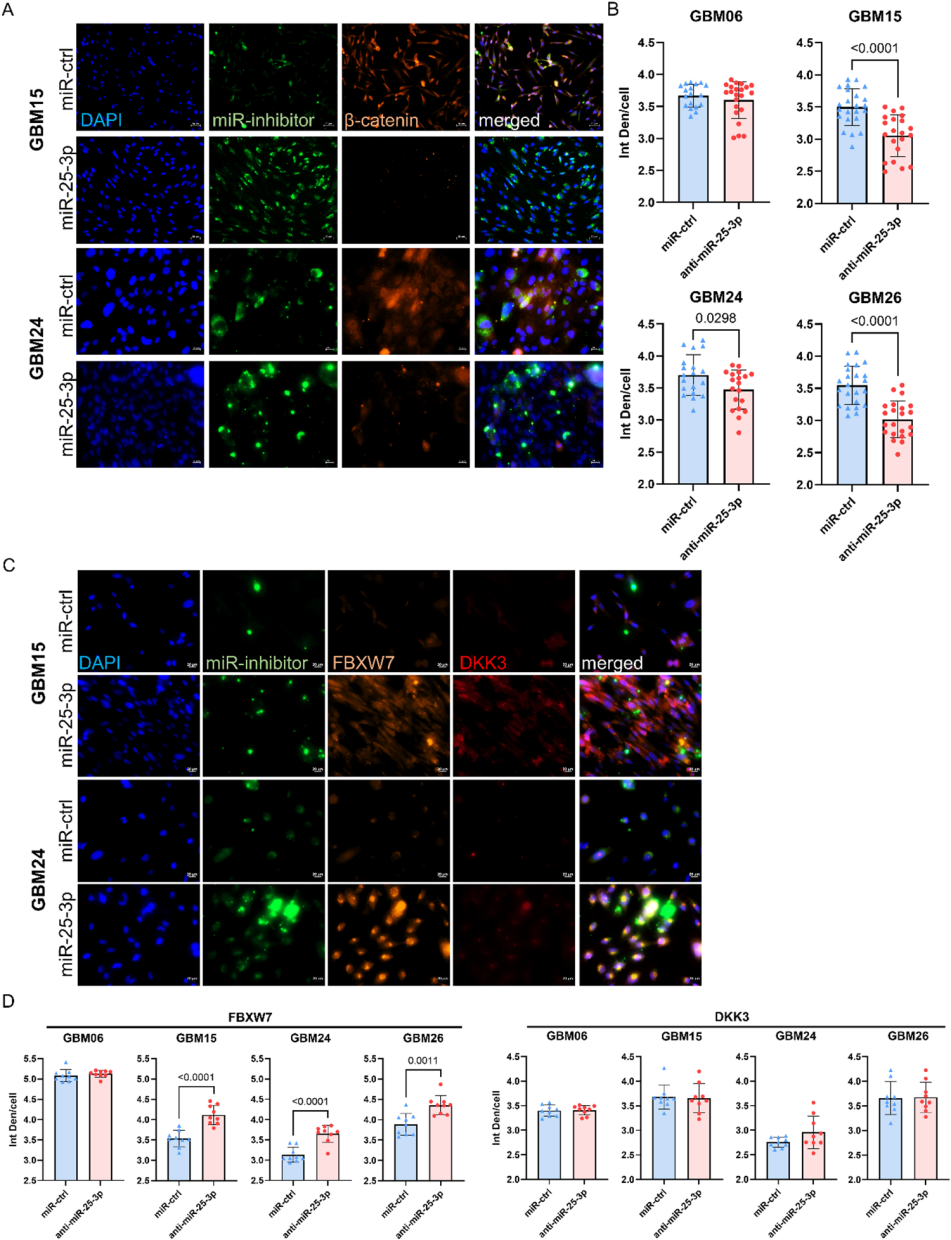
Inhibition of miR-25-3p downregulates β-catenin expression and upregulates key tumor suppressor genes. **A** Representative immunofluorescence images of GBM15 and GBM24 cells stained for β-catenin after miR-inhibitor incubation. Scale bar = 20 μm. **B** Quantification of β-catenin expression after incubation with miR-25-3p inhibitor or miR-control in GBM06, GBM15, GBM24, GBM26. Mean ± SD, *n* = 3 independent experiments with three technical replicates. Unpaired t test. **C** Representative immunofluorescence images of GBM15 and GBM24 cells stained for FBXW7 and DKK3 after miR-inhibitor incubation. Scale bar = 20 μm. **D** Quantification of FBXW7 (left) and DKK3 (right) expression after incubation with miR-25-3p inhibitor or miR-control in GBM06, GBM15, GBM24, GBM26. Mean ± SD, *n* = 3 independent experiments with two technical replicates. Unpaired t test

To investigate whether the TMZ sensitizing effect of miR-25-3p inhibition is mediated through β-catenin downregulation, we analyzed β-catenin expression following treatment with a miR-25-3p inhibitor. A non-selective miRNA-control was also included. In three of the four tested cell lines (GBM15, GBM24_4, and GBM26), β-catenin expression was significantly reduced following miR-25-3p knockdown (*p* < 0.0001 to *p* < 0.05; Fig. 3A, B). In contrast, GBM06 – previously shown to have poor miRNA-inhibitor uptake – displayed no significant change in β-catenin levels. These findings support a mechanistic link between effective miR-25-3p inhibition and β-catenin suppression, consistent with the observed TMZ sensitization (Fig. 2E, G). To further elucidate the mechanism underlying this effect, we examined the expression of two validated miR-25 targets: FBXW7 and DKK3, both known to negatively regulate β-catenin signaling. Immunofluorescence analysis revealed upregulation of FBXW7 in GBM cell lines exhibiting reduced β-catenin expression and enhanced TMZ sensitivity (Fig. 3C, D). However, DKK3 expression remained unchanged, suggesting that miR-25-3p contributes to chemoresistance by suppressing key β-catenin inhibitors. This, in turn, may reduce β-catenin primarily through FBXW7 upregulation.

### miR-25-3p Inhibition Reduces Invasiveness

**Fig. 4:**
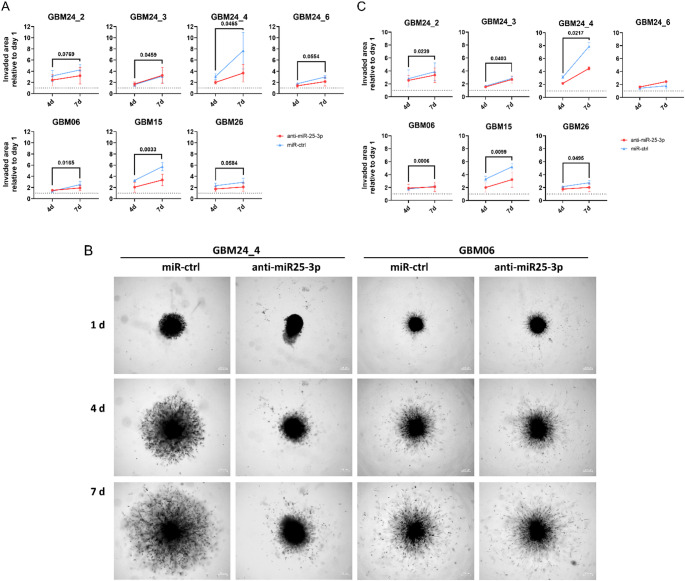
miR-25-3p inhibition reduces invasion in selected GBM cell lines, independently of TMZ treatment. **A** The graph shows the quantified invaded area relative to day 1 for seven cell lines after day 4 and 7. Mean ± SD, *n* = 3 independent experiments with three technical replicates. Two way ANOVA. **B** Representative images of invasion assay of cells after miR-inhibitor incubation on day 1, 4, and 7 after spheroids were embedded in matrigel. **C** The graph shows the quantified invaded area relative to day 1 for seven cell lines after day 4 and 7. Cell where treated with 10 µM TMZ at the time of embedding in the matrigel. Mean ± SD, *n* = 3 independent experiments with three technical replicates. Two-way ANOVA

To investigate the role of miR-25-3p in regulating invasive behavior – a key factor contributing to treatment resistance and recurrence in GBM – we conducted in vitro invasion assays following miR-25-3p inhibition across all cell lines. Consistent with our previous findings, GBM15 showed a significant reduction in invasiveness upon miR-25-3p inhibition (*p* < 0.01), while GBM26 exhibited a moderate decrease. In contrast, invasion of GBM06 remained unaffected, again correlating with its previously observed low inhibitor uptake (Fig. 4A, B). To further explore intratumoral heterogeneity, we analyzed the four subclones of GBM24 (GBM24_2, _3, _4, and _6). Among these, GBM24_4, which displayed the strongest baseline invasiveness out of the four subclones, exhibited a statistically significant decrease in invasive capacity (*p* < 0.05) after miR-25-3p inhibition, and GBM24_2 displayed a similar trend. In contrast, invasiveness of GBM24_3 and GBM24_6 was largely unaffected by miR-25-3p inhibition, highlighting the functional diversity within tumor subpopulations. To evaluate potential combinatorial effects, invasion assays were repeated with concurrent TMZ treatment (Fig. 4B, C). Across all tested cell lines, no additional reduction in invasiveness beyond that induced by miR-25-3p inhibition alone was observed. This suggests that the pro-invasive role of miR-25-3p is independently of TMZ-mediated DNA damage responses.

### miR-25-3p Inhibition Reshapes the Transcriptional Landscape of GBM Cells

**Fig. 5:**
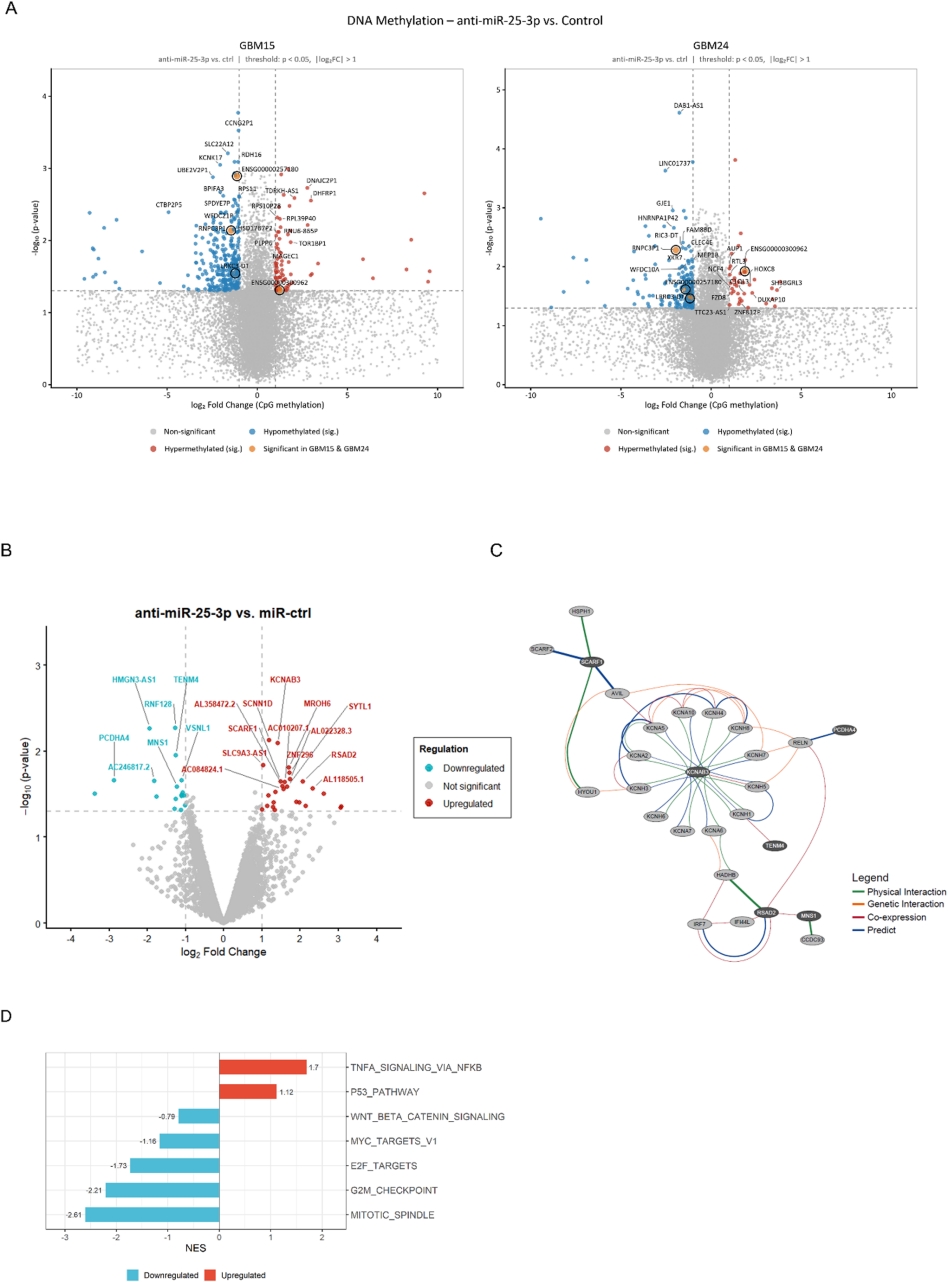
Inhibition of miR-25-3p deregulated proteins with a focus on immune regulation. **A** Volcano plot showing significantly hyper- and hypomethylated genes in GBM15 (left) and GBM24 (right) after miR-25-3p inhibition (|log₂ fold change| >1 and padj < 0.05), *n* = 2 cell lines with three biological replicates. **B** Volcano plot showing significantly deregulated genes in GBM24 after miR-25-3p inhibition (|log₂ fold change| >1 and padj < 0.05). **C** STRING network analysis was performed to visualize the interaction of deregulated proteins based on the deregulated DEGs. The type of interaction is marked by color (green = physical interaction, orange = genetic interaction, red = co-expression, and blue = predicted interaction), weight of the interaction is given by line size and deregulated proteins are highlighted, *n* = 1 cell line with three biological replicates. **D** Graph shows deregulated gene sets in GBM24 after miR-25-3p inhibition (NES = normalized enrichment score; dashed lines at NES +/- 1.5), *n* = 1 cell line with three biological replicates

Given the pronounced effects of miR-25-3p inhibition on the GBM cell phenotype, we performed DNA methylation profiling in miR-25-3p–inhibited GBM15 and GBM24 cells compared with controls. This analysis identified 600 differentially methylated genes (|log₂ fold change| > 1; adjusted *p* < 0.05). In GBM15, 306 genes were hypomethylated and 80 hypermethylated, whereas in GBM24, 176 genes were hypomethylated and 38 hypermethylated (Fig. 5A). Three genes (*RNPC3P1*, *LRRC3-DT*, *ENSG00000257180*) were significantly hypomethylated in both cell lines, and one gene (*ENSG00000300962*) was consistently hypermethylated; all represent pseudogenes or lncRNAs (long non-coding RNAs) with currently uncharacterized functions.

Among the hypomethylated genes of interest were *RDH16*, implicated in tumor cell stemness (Hu et al. 2017), and *XKR7*, a member of the XK protein family associated with apoptosis regulation (Suzuki et al. 2014). Notably, hypermethylated genes included *FZD8*, a regulator of cancer invasiveness *via* WNT/β-catenin signaling (Ueno et al. 2013), as well as *AUP1* and *MAGEC1*, both linked to immune regulatory processes (Mattila et al. 2011; Chen et al. 2022). *C1QL3*, involved in synapse formation and maintenance and glucose homeostasis, was also hypermethylated, indicating cellular dedifferentiation (Caro et al. 2025). To further characterize transcriptional changes, we performed RNA sequencing in miR-25-3p–inhibited GBM24 cells. We identified 50 differentially expressed genes (|log₂ fold change| > 1; adjusted *p* < 0.05), including 30 upregulated and 20 downregulated genes (Fig. 5B). Upregulated genes comprised *RSAD2*, *SCARF1*, and *KCNAB3*, which are implicated in immune regulation and may contribute to the observed anti-tumor effects. Conversely, downregulated genes included *PCDHA4*, involved in neuronal cell–cell interactions; *MNS1*, associated with PI3K/AKT-mediated metastasis; *RNF128*, an oncogenic E3 ubiquitin ligase; and *TENM4*, linked to stemness and migration. STRING network analysis revealed functional interactions among several of these genes (Fig. 5C). In addition, a GSEA analysis revealed several dysregulated gene sets after the miR-25-3p inhibition. Among the downregulated gene sets were genes directly linked to WNT/β-catenin signaling and genes representing downstream targets of this pathway (e.g. *MYC* and *E2F*) (Katoh and Katoh 2007; Yang et al. 2017). Moreover, several gene sets involved in proliferation, including G2M_Checkpoint and Mitotic_Spindle (Jiang et al. 2015; Ogita et al. 2018) were downregulated, whereas gene sets linked to stress responses, such as P53_Pathway and TNFA_Signaling_Via_NFκB (Mitchell et al. 2016; Tian et al. 2024) were upregulated (Fig. 5D). Collectively, these data indicate that miR-25-3p inhibition is associated with epigenetic and transcriptional reprogramming that align with a less aggressive and less invasive phenotype, accompanied by impaired cellular communication.

## Discussion

In this study, we profiled eight specific miRNAs in 50 primary GBM samples and seven matched patient-derived 2D and 3D in vitro models. Inclusion of both dimensional platforms provides a more physiologically relevant context for interrogating miRNA-mediated regulation. To address intratumoral heterogeneity, we focused on four cell lines derived from the same primary tumor, minimizing inter-patient variability while capturing intrinsic cellular diversity. Among the eight miRNAs examined, miR-135a-5p and miR-135b-5p were significantly upregulated in the *MGMT*-methylated group. This is notable given their reported tumor-suppressive roles in GBM (Gomez Zubieta et al. 2017; Istiqamah et al. 2023) and the established link between *MGMT* promoter methylation and favorable prognosis (Hegi et al. 2005), suggesting that miR-135 family members may contribute to a less aggressive tumor phenotype or influence treatment sensitivity. Nearly half of the profiled miRNAs demonstrated a trend toward higher expression in *TP53*-mutant cases as potential compensatory or oncogenic regulatory dynamics. Among these, miR-10b-5p was significantly upregulated, which aligns with its known oncogenic role across cancers(Ladeiro et al. 2008; Sasayama et al. 2009; Chai et al. 2010; Yamamoto 2012). In GBM (Sasayama et al. 2009), miR-10b-5p promotes invasion, proliferation, and resistance to apoptosis. Given that the *TP53*-miRNA axis is a critical regulatory hub in gliomagenesis, our findings indicate that loss of *TP53* function may increase the expression of specific oncogenic miRNAs, promoting tumor progression. Although the prognostic relevance did not reach statistical significance in our cohort, most likely due to the limited sample size, future studies with larger patient numbers will help to clarify this effect. Conversely, elevated expression of miR-129-3p and miR-129-5p was associated with improved overall survival, supporting their tumor-suppressive role (Ma et al. 2019; Boicean et al. 2023).

Our analyses of tumor localization revealed significant differences in the expression of miR-10b-5p and miR-135b-5p in regions of the parietal lobe and deeper structures, as opposed to the temporal and frontal lobes. Moreover, all analyzed regions were characterized by a consistent high expression of miR-25-3p, pointing strongly to a potential high relevance of this oncogenic miRNA. This highlights the potential influence of localization on miRNA expression in GBM, as further highlighted by the spatio-temporal study of brain miRNAs conducted by Engel et al. (2025). Evaluation of four miRNAs across seven patient-derived GBM cell lines cultured in both 2D and 3D revealed consistently higher expression in 3D spheroids, suggesting upregulation in environments with enhanced cell–cell and cell-matrix interactions. Supporting this, our EV analysis demonstrated that they contained higher levels of specific miRNAs than the originating cells. This finding is consistent with growing evidence that miRNAs mediate intercellular communication, potentially via EVs that transport both RNAs and proteins (Sepúlveda et al. 2023).

We next focused on miR-25-3p due to its consistently strong expression and reported functional relevance in GBM (Wang et al. 2021). Using a complementary antisense inhibitor, we effectively downregulated miR-25-3p. Control experiments confirmed minimal off-target toxicity, and a double-application protocol enhanced intracellular uptake, sustaining miR-25-3p suppression for at least six days. Uptake efficiency varied across cell lines, indicating that the observed effects were primarily due to specific miR-25-3p inhibition. Notably, miR-25-3p inhibition re-sensitized four of the seven analyzed patient-derived cell lines to TMZ, with the effect correlating with uptake efficiency. These results support a mechanistic link between miR-25-3p and chemoresistance (Wang et al. 2021), and suggest that its suppression may partially restore TMZ responsiveness in GBM. Interestingly, among the four GBM24-derived cell lines, only two showed increased TMZ sensitivity upon miR-25-3p inhibition. This underscores the pronounced intratumoral heterogeneity in GBM—even among genetically related subclones—and the challenges it poses for treatment. Consistent results in 3D cultures further support a role for miR-25-3p in mediating TMZ resistance. These findings underscore the importance of patient- and subclone-specific strategies for miRNA-based interventions and the need for heterogeneity-aware models in preclinical drug testing. Assessment of cellular behavior showed that miR-25-3p inhibition reduced invasiveness, particularly in the most aggressive cell lines, implicating a role for miR-25-3p in promoting invasion, potentially *via* cytoskeletal remodeling or extracellular matrix degradation. Notably, TMZ treatment did not significantly influence invasion, suggesting that the invasive properties of these cells are largely independent of chemotherapeutic exposure.

To explore the mechanisms underlying enhanced TMZ sensitivity upon miR-25-3p inhibition, we identified re-expression of FBXW7 and partially DKK3, both validated as direct miR-25-3p targets and independently implicated in TMZ resistance (Fujihara et al. 2018; Wang et al. 2021; Proto et al. 2022). DKK3 and FBXW7 converge on the WNT/β-catenin signaling pathway, which is frequently dysregulated in cancer (Moon et al. 2004; Reya and Clevers 2005; Klaus and Birchmeier 2008; Zhan et al. 2017). In GBM, this interaction promotes autophagy-mediated TMZ resistance (Yun et al. 2020), in part mediated by WNT/β-catenin–dependent transcription of anti-apoptotic proteins such as MCL-1 and Aurora B, which support cell survival and G2/M checkpoint progression (Krajewski et al. 1995; Liu et al. 2003; Dutta-Simmons et al. 2009; Ma et al. 2019; Sancho et al. 2022)—mechanisms frequently co-opted in TMZ-resistant GBM. By targeting DKK3 and FBXW7, miR-25-3p may sustain β-catenin activity, thereby promoting transcription of anti-apoptotic effectors such as MCL-1 and Aurora B. Hence, inhibition of the regulatory miR-25-3p in GBM cell lines is associated with reduced β-catenin levels. The only exception was GBM06, consistent with lower uptake of the miRNA-specific inhibitor and maintained resistance to TMZ in both 2D and 3D culture models. These results suggest a direct relationship between miR-25-3p inhibitor uptake and β-catenin downregulation, further supported by increased FBXW7 expression following miR-25-3p inhibition in the same cell lines that exhibited reduced β-catenin levels. Again, GBM06 deviated from this pattern, displaying no significant change in FBXW7. Notable, baseline FBXW7 levels in GBM06 were higher than in the other cell lines, which may partly explain the absence of further induction after inhibitor treatment. Thus, miR-25-3p appears to play a less prominent role in this particular model, aligning with its relatively low expression – especially in 3D cultures and cell-derived EVs. Nevertheless, this difference is unlikely to fully account for the low response, suggesting that additional, yet unidentified, mechanisms may be involved.

Our integrative epigenetic and transcriptomic analyses indicate that miR-25-3p inhibition extends beyond post-transcriptional regulation and induces coordinated reprogramming of oncogenic signaling networks in GBM cells. DNA methylation profiling revealed widespread epigenetic remodeling in both GBM15 and GBM24, with a predominance of hypomethylated genes, although limited overlap between models likely reflects intertumoral heterogeneity. Notably, *FZD8*, a key activator of WNT/β-catenin signaling (Ueno et al. 2013), was hypermethylated following miR-25-3p inhibition. In addition, transcriptome-wide gene set enrichment analysis identified multiple dysregulated pathways following miR-25-3p inhibition, including gene sets directly related to WNT/β-catenin signaling as well as downstream transcriptional genes associated with pathway effectors such as *MYC* and *E2F* (Katoh and Katoh 2007; Yang et al. 2017). Together with our independent observation of reduced β-catenin levels, this finding supports functional attenuation of WNT signaling, an axis critically implicated in glioblastoma invasiveness, stemness, and therapeutic resistance (Klaus and Birchmeier 2008). Additional methylation changes in genes such as *RDH16*, linked to tumor cell stemness, and *XKR7*, associated with apoptosis regulation, further suggest modulation of cellular plasticity and survival pathways (Suzuki et al. 2014; Hu et al. 2017) Hypermethylation of immune-related genes, including *AUP1* and *MAGEC1* (Mattila et al. 2011; Chen et al. 2022), points toward epigenetic remodeling of tumor–immune interactions, reinforcing the notion that miR-25-3p may act at the interface between tumor-intrinsic signaling and immune regulation.

Consistently, transcriptomic profiling demonstrated that miR-25-3p inhibition reshapes gene networks involved in immune modulation, cellular communication, and tumor aggressiveness. Genes implicated in anti-tumor immune responses (Patten et al. 2020; Ebrahimi et al. 2021; Li et al. 2023), including *RSAD2*, *SCARF1*, and *KCNAB3*, were upregulated, suggesting that suppression of miR-25-3p may enhance anti-tumor immune responses or promote a proinflammatory tumor microenvironment. This is consistent with emerging evidence that miRNAs serve as key modulators at the interface between tumor cells and immune surveillance. Conversely, downregulation of *PCDHA4*, a member of the protocadherin alpha cluster critical for neuronal connectivity (Yi et al. 2021), may reflect altered cell–cell interactions within the tumor niche. Moreover, suppression of *MNS1*, a promoter of metastasis via PI3K/AKT activation, *RNF128*, an oncogenic E3 ligase (Wang et al. 2025), and *TENM4*, associated with stemness and migration, further support the notion that miR-25-3p inhibition reprograms GBM cells toward a less aggressive and less invasive phenotype. The concurrent deregulation of multiple long non-coding RNAs adds an additional layer of regulatory complexity, with their precise functional roles yet to be defined. In summary, our findings provide compelling evidence for the regulatory role of the oncogenic miR-25-3p in GBM, with its inhibition sensitizing cells to TMZ. Mechanistically, this effect is driven by upregulation of *FBXW7* and subsequent downregulation of β-catenin. These insights not only deepen our understanding of miR-25-3p’s functional significance in GBM pathobiology but also highlight its potential as a therapeutic target. Further studies, particularly in patient-derived organoids, will be critical to determine the translational relevance of these findings and to assess the feasibility of miR-25-3p–targeted interventions in clinical settings.

## Data Availability

The datasets generated during and/or analyzed during the current study are available from the corresponding author on reasonable request. The amplicon sequencing data of this study are available in the Gene Expression Omnibus repository at https://www.ncbi.nlm.nih.gov/sra (Accession No. PRJNA1439168 and PRJNA1439717).

## Abbreviations

miRNA: microRNA
GBM: Glioblastoma
TMZ: Temozolomide
EV: Extracellular vesicle
FBXW7: F-box/WD repeat-containing protein 7
DKK3: Dickkopf-3
DEG: Differentially expressed genes
GO: Gene ontology
GSEA: Gene set enrichment analysis
Int den/cell: Integrated dencity per cell
NES: Normalized enrichment score
